# Supplementary material for: Diversity and its decomposition into variety, balance and disparity
Source: R Soc Open Sci. 2019 Jul 17;6(7):190452. doi: 10.1098/rsos.190452 (PMC6689592; doi:10.1098/rsos.190452)
Supplement: Supplementary material [file rsos190452supp1.pdf]

Supplementary material
Diversity and its decomposition into variety, balance and disparity

Alje van Dam*

Copernicus Institute for Sustainable Development, Utrecht University
Centre for Complex Systems Studies (CCSS), Utrecht University

May 29, 2019

Contents

A Hill numbers and entropy **2**

B Properties from Leinster & Cobbold **3**

C Multiple feature sets **7**

D Aggregation **8**

*Email: A.vanDam@uu.nl

A Hill numbers and entropy

In the definition of diversity we rely on the concept of Hill numbers, following [1] and [2]. The Hill number of order q is given by the reciprocal of a generalized mean of the relative frequencies. The generalized weighted mean of the relative frequencies of types is given by

$$^{q}\bar{p} = \sqrt[q-1]{\sum_i p_i p_i^{q-1}}, \tag{1}$$

where the weights are given by the relative frequencies p_i. The parameter q determines which mean is considered. For example, $^{0}\bar{p}$ denotes the Harmonic mean, $^{1}\bar{p}$ the geometric mean and for $^{2}\bar{p}$ the arithmetic mean [1]. The Hill number of order q measures the diversity of types as the reciprocal of the mean

$$^{q}D(S) = \frac{1}{^{q}\bar{p}} = \left(\sum_i p_i^q\right)^{\frac{1}{1-q}}.$$

The parameter q determines how heavily the average weights common or rare species. Values of $q > 1$ weigh more heavily types with high relative frequency, and values of $q < 1$ weigh more heavily the presence of types with small relative frequency. The minimal value of $q = 0$ considers every type to contribute equally to the mean, regardless of its relative frequency. For $q = 0$ the diversity is given by

$$^{0}D(S) = \sum_i 1 = n$$

and gives simply a count of the number of types in S. The Hill number of order 0 is thus a measure of *variety*, which is also known as species richness in ecology.

For $q = 2$, one obtains

$$^{2}D(S) = \sum_i \frac{1}{p_i^2},$$

which relates directly to Simpson's index of concentration and the Gini-index [2].

In general, the Hill numbers are related to the Rényi entropy [4] by ${}^qD(S) = e^{{}^qH(X)}$, where

$$^qH(X) = \frac{1}{1-q} \log\left(\sum_i p_i^q\right).$$

The Shannon entropy arises as a special case when taking the limit of $q \to 1$. This corresponds to the unique Hill number that does not favor either rare or common types and is given by

$$D(S) = \lim_{q\to 1} {}^qD(S) = e^{-\sum_i p_i \log(p_i)} = e^{H(X)}.$$

The relationship between Hill numbers and entropies described above tell us how to transform measures of uncertainty, given by entropies in units of bits or nats, into measures of diversity, given in units of the 'effective number of types'. The more uncertain one is about the type of a randomly sampled element from S (i.e. the higher ${}^qH(X)$), the more diverse the set S in considered to be.

B Properties from Leinster & Cobbold

In their introduction of a diversity measure that takes into account disparity by including pairwise similarities between types, Leinster & Cobbold [3] show that their measure satisfies nine properties that 'encode basic scientific intuition' that every diversity measure should satisfy. The nine properties are divided into three categories: partitioning properties, elementary properties, and similarity properties. In this section it is shown that the properties posed in [3] also hold for the number of compositional units $D_\beta(S')$.

We follow the notation as introduced in the main text: a collection of features $i \in S$, a collection of types $j \in S'$, and their corresponding random variables X, Y and XY with probabilities $p_i = P(X = i)$, $p_j = P(Y = j)$, and $p_{ij} = P(X = i, Y = j)$ respectively.

Partitioning

Effective number: the diversity of a community of n equally abundant, totally dissimilar types is n.

Note that when all types are totally dissimilar, there is no uncertainty about the type j of an element given that one knows its feature i. That is, for

every feature i we have that $p_{j|i} = 1$ for one specific type j. This implies that $H(Y|X) = -\sum_i p_i \sum_j p_{j|i} \log(p_{j|i}) = 0$, so that

$$\begin{aligned} MI(X,Y) &= H(X) + H(Y) - H(XY) \\ &= H(Y) - H(Y|X) \\ &= H(Y). \end{aligned}$$

Then

$$D_\beta(S') = e^{MI(X,Y)} = e^{H(Y)} = D(S').$$

Hence for totally dissimilar types the number of compositional units reduces to the effective number of types. In particular, for equally abundant types we have $D_\beta(S') = e^{H(Y)} = n$.

Modularity: if a collection of types consists of multiple non-overlapping sub-collections of types, for which types in different sub-collections are totally dissimilar, then the total diversity is entirely determined by the size and diversity of every sub-collection.

We can implement the sub-collections by adding a third label k to every element, which denotes the sub-collection $k \in S''$ it belongs to. Hence, we now have elements with labels i, j, k, where i denotes a feature, j denotes a type, and k denotes the sub-collection. Further introducing the corresponding random variable Z, this defines probabilities $p_{ijk} = P(X = i, Y = j, Z = k)$. Since sub-collections are non-overlapping, there is no uncertainty about the sub-collection k of an element given that one know its type j, so that $H(Z|Y) = 0$. Furthermore, since types from different sub-collections are totally dissimilar, sub-collections do not share any features, so there is no uncertainty about the sub-collection k of an element given that one knows its feature i, so $H(Z|X) = 0$. These properties imply that $H(YZ) = H(Y)$ and $H(XZ) = H(X)$. Defining

$$MI(X,Y|Z) = \sum_k p_k \sum_{ij} p_{ij|k} \log\left(\frac{p_{ij|k}}{p_{i|k} p_{j|k}}\right),$$

we can then write

$$\begin{aligned}MI(X,Y|Z) &= H(X|Z) + H(Y|Z) - H(XY|Z)\\ &= H(XZ) - H(Z) + H(YZ) - H(Z) - H(XY) + H(Z)\\ &= H(X) + H(Y) - H(XY) - H(Z)\\ &= MI(X,Y) - H(Z)\end{aligned}$$

so that

$$MI(X,Y) = MI(X,Y|Z) + H(Z).$$

Taking the exponential, this shows how the total number of compositional units of types S' relates to the number of compositional units in each sub-collection k, their relative size p_k, and the effective number of sub-collections $D(S'')$:

$$\begin{aligned}D_\beta(S') &= e^{MI(X,Y)}\\ &= e^{MI(X,Y|Z)+H(Z)}\\ &= e^{\sum_k p_k MI(X,Y|k)+H(Z)}\\ &= D(S'')\prod_k D_\beta(S'_k)^{p_k},\end{aligned} \tag{2}$$

where $D(S'') = e^{H(Z)}$ denotes the effective number of sub-collections.

Replication: if m non-overlapping sub-collections are of equal size and diversity d, the diversity of the whole collection is given by md.

Using (2), it is easily seen that if the number of compositional units in every sub-collection is d, and there are m sub-collections with relative size $\frac{1}{m}$, we have

$$D_\beta(S') = m\prod_k d^{\frac{1}{m}} = md.$$

Elementary

Symmetry: diversity is independent of the order of the listing of types.

This property follows directly from the properties of the Shannon entropy.

Absent types: diversity is unchanged by adding a type of zero abundance.

This property follows directly from the properties of the Shannon entropy.

Identical types: for two identical types, merging the types leaves diversity unchanged.

Recall that XY is defined as the random variable with probabilities $p_{ij} = P(X = i, Y = j)$, where $i \in S$ and $j \in S'$. For two identical types j' and j'', we have that $p_{i|j'} = p_{i|j''}$ since they have an identical distribution over features.

Define a random variable $X\tilde{Y}$ in which j' and j'' are merged, i.e. $\tilde{p}_{ij'} = P(X = i, \tilde{Y} = j) = p_{ij'} + p_{ij''}$, $\tilde{p}_{ij''} = 0$ and $\tilde{p}_{ij} = p_{ij}$ for all $j \neq j', j''$. Then

$$\begin{aligned} MI(X,Y) &= \sum_{ij, j\neq j',j''} p_{ij} \log\left(\frac{p_{i|j}}{p_i}\right) + \sum_i p_{ij'} \log\left(\frac{p_{i|j'}}{p_i}\right) + \sum_i p_{ij''} \log\left(\frac{p_{i|j''}}{p_i}\right) \\ &= \sum_{ij, j\neq j',j''} p_{ij} \log\left(\frac{p_{i|j}}{p_i}\right) + \sum_i (p_{ij'} + p_{ij''}) \log\left(\frac{p_{i|j'}}{p_i}\right) \\ &= MI(X, \tilde{Y}). \end{aligned}$$

Hence, $D_\beta(S') = D_\beta(\tilde{S}')$, so merging identical types does not affect the number of compositional units.

Effect of similarity on diversity

Monotonicity: when similarity between types is increased, diversity decreases.

Although we do not have an explicit measure of pairwise similarity between types, similarity in our framework is given by the (average) overlap of features between types. This overlap may increase in two ways: either the total diversity of features $D_\gamma(S)$ decreases while the average within-type diversity $D_\alpha(S)$ remains constant, or the average within-type diversity $D_\alpha(S)$ increases while the total diversity of features $D_\gamma(S)$ remains constant. From the definition of the number of compositional units $D_\beta(S') = \frac{D_\gamma(S)}{D_\alpha(S)}$ it follows that in both cases the number of compositional units decreases.

Naive model: when similarities are ignored, diversity is greater or equal than when similarities are taken into account.

This follows directly from the definitions of $D_\beta(S')$ (which takes into account disparity) and $D(S')$ (which does not take into account disparity), and the known property that $MI(XY) \le H(Y)$. This leads to

$$D(S') = e^{H(Y)} \ge e^{MI(X,Y)} = D_\beta(S').$$

Range: the diversity of a collection of n types is between 1 and n.

We have that $0 \le MI(XY) \le H(Y) \le \log(n)$. Taking exponentials, this gives $1 \le D_\beta(S') \le n$.

C Multiple feature sets

This section elaborates on the results given in the main text on diversity when taking into account two feature sets, described by random variables X and Y. The feature pairs are then described by the joint distribution $p_{ij} = P(X = i, Y = j)$. Using the simple additive properties of information-theoretic quantities, we show some simple results regarding diversities. The calculations are easily verified by considering the Venn diagrams in Figure 1.

Here, we rewrite the diversity of types corresponding to random variable Z given the overlap among a pair of features given by random variables X and Y as

$$\begin{aligned} D_\beta^{XY}(S') &= e^{MI(XY,Z)} \\ &= e^{H(XY)-H(XY|Z)} \\ &= e^{H(X)+H(Y)-MI(X,Y)-H(X|Z)-H(Y|Z)+MI(X,Y|Z)} \\ &= e^{MI(X,Z)+MI(Y,Z)-MI(X,Y)+MI(X,Y|Z)}, \end{aligned} \tag{3}$$

where we used that $H(XY) = H(X) + H(Y) - MI(X,Y)$ and $H(XY|Z) = H(X|Z) + H(Y|Z) - MI(X,Y|Z)$. From this, it becomes clear that the diversity becomes lower as the features X and Y have a larger dependence, i.e. are more correlated, as indicated by a large value of $MI(X,Y)$.

In the special case that features X and Y share no information, i.e.

$MI(X,Y)=0$, we have

$$D_\beta^{XY}(S') = e^{MI(XY,Z)} \tag{4}$$
$$= e^{MI(X,Z)+MI(Y,Z)}$$
$$= D_\beta^X(S')D_\beta^Y(S').$$

Hence, for independent feature sets the diversities are multiplicative.

D Aggregation

Here we consider the types described by random variable Z to be composed of features described by random variable Y, and the features Y themselves have features described by a random variable X (this reflects the situation described in the modularity property in Section B, where Z denotes the sub-collections, Y denotes the types, and X denotes the features). Hence the links between types and features are given by the joint probability distribution p_{jk}, and the links between features and 'sub-features' by a joint distribution p_{ij}. When the joint probabilities p_{ij} are independent of the joint probabilities p_{jk}, we have $p_{ijk} = p_{ij}p_{k|j} = p_{i|j}p_{k|j}p_j$. In other words, the random variables Z and X are conditionally independent given Y, which means that $MI(X,Z|Y)=0$. The diversity given feature pairs XY can then be rewritten as

$$D_\beta^{XY}(S') = MI(XY,Z) \tag{5}$$
$$= e^{H(Z)-H(Z|XY)}$$
$$= e^{H(Z)-(H(ZX|Y)-H(X|Y))}$$
$$= e^{H(Z)-H(Z|Y)} = e^{MI(Z,Y)},$$

where we used that $MI(X,Z|Y)=0$ implies that $H(XZ|Y)-H(X|Y)=H(Z|Y)$. In other words, considering X is superfluous when considering the diversity of Z in terms of features XY.

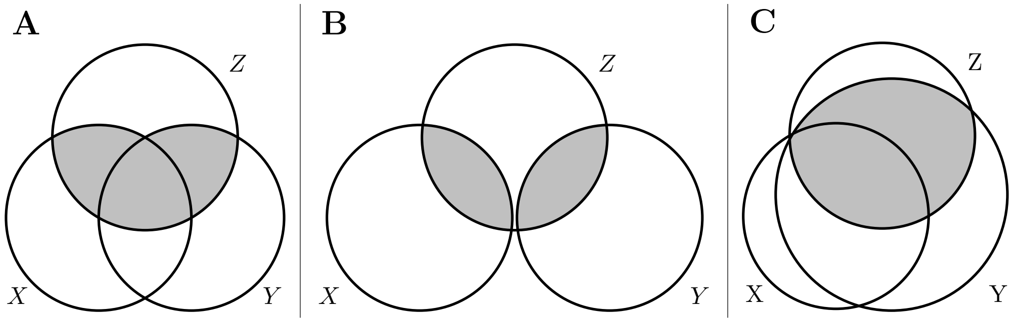

Figure 1: The entropies and mutual information can be represented using Venn diagrams, where each circle corresponds to the entropy $H(X)$ of the associated random variable X. The intersection of the two circles associated to X and Y represents the mutual information $MI(X, Y)$, and their union represents the joint entropy $H(XY)$. The conditional entropy $H(X|Y)$ is given by subtracting the intersection from the total uncertainty $H(X)$. **A** shows the mutual information $MI(XY, Z)$ from equation (3). The diversity of variable Z given the overlap in features XY is given by the exponential of the shaded area. **B** shows the special case of (4) in which the features X and Y are independent, i.e. $MI(X, Y) = 0$. From the figure it is clear that $MI(XY, Z) = MI(X, Z) + MI(Y, Z)$, such that associated diversity in this case is multiplicative. **C** shows the case of (5) in which Z and X are conditionally independent on Y, i.e. $MI(Z, X|Y) = 0$. In this case, taking into account features X becomes irrelevant in computing diversity of Z given feature pairs XY.

References

[1] M. O. Hill. Diversity and Evenness: A Unifying Notation and Its Consequences. *Ecology*, 54(2):427–432, mar 1973. doi:10.2307/1934352.

[2] L. Jost. Entropy and diversity. *Oikos*, 113(2):363–375, may 2006. doi:10.1111/j.2006.0030-1299.14714.x.

[3] T. Leinster and C. A. Cobbold. Measuring diversity: the importance of species similarity. *Ecology*, 93(3):477–489, mar 2012. doi:10.1890/10-2402.1.

[4] A. Renyi and A. Rényi. On Measures of Entropy and Information. In *Proceedings of the Fourth Berkeley Symposium on Mathematical Statistics and Probability, Volume 1: Contributions to the Theory of Statistics*, volume 1 of *Fourth Berkeley Symposium on Mathematical Statistics and*

Probability, pages 547–561, Berkeley, Calif., 1961. University of California Press. URL https://projecteuclid.org/euclid.bsmsp/1200512181.
